# Distal Anastomotic New Entries After Acute Type A Aortic Dissection Surgery: A Retrospective Cohort Study

**DOI:** 10.1093/icvts/ivag262

**Published:** 2026-09-25

**Authors:** Stefan R van Dinter, Pim S van Sambeeck, Nesar A Hasami, Amir H Sadeghi, Michel W A Verkroost, Tychon E A Geeraedts, Jan Jaap Janssen, Foeke J H Nauta, Nabil Saouti, Robin H Heijmen

**Affiliations:** Department of Cardiothoracic Surgery, Radboud University Medical Centre, 6525 GA Nijmegen, Netherlands; Department of Cardiothoracic Surgery, Radboud University Medical Centre, 6525 GA Nijmegen, Netherlands; Department of Cardiothoracic Surgery, Radboud University Medical Centre, 6525 GA Nijmegen, Netherlands; Department of Cardiothoracic Surgery, Radboud University Medical Centre, 6525 GA Nijmegen, Netherlands; Department of Cardiothoracic Surgery, Radboud University Medical Centre, 6525 GA Nijmegen, Netherlands; Department of Radiology, Radboud University Medical Centre, 6525 GA Nijmegen, Netherlands; Department of Radiology, Radboud University Medical Centre, 6525 GA Nijmegen, Netherlands; Department of Cardiothoracic Surgery, Radboud University Medical Centre, 6525 GA Nijmegen, Netherlands; Department of Cardiothoracic Surgery, Radboud University Medical Centre, 6525 GA Nijmegen, Netherlands; Department of Cardiothoracic Surgery, Radboud University Medical Centre, 6525 GA Nijmegen, Netherlands

**Keywords:** DANE, distal anastomotic new entry, aortic dilatation, aortic dissection, distal aortic reintervention, negative aortic remodelling

## Abstract

**Objective:**

Distal anastomotic new entry (DANE) after acute type A aortic dissection repair may contribute to adverse downstream aortic remodelling. This study evaluated the incidence of postoperative DANE and its association with distal aortic growth, reintervention, and clinical outcomes.

**Design:**

All consecutive patients undergoing type A aortic dissection repair with residual downstream dissection (DeBakey I) between 2011 and 2024 were retrospectively analyzed. Computed tomography angiography was systematically reviewed for DANE using 2-plane distal anastomosis alignment. Longitudinal descending thoracic aortic maximum diameters were analyzed using linear mixed-effects models. Univariable logistic regression, Kaplan-Meier estimates, and Cox proportional hazards analyses were performed.

**Results:**

A total of 164 patients were included, of whom 77 (47.0%) had postoperative DANE. Patients with DANE demonstrated larger distal aortic diameters over time compared with patients without DANE, while baseline diameters were similar (37.6 vs 37.3, *P* = .77). At 3-5 years, estimated maximum descending thoracic aortic diameter was 51.6 vs 42.7 mm, respectively (*P* < .001). DANE was associated with ≥10-mm downstream aortic growth, ≥55-mm maximum diameter, and reintervention during 5-year follow-up (combined odds ratio 5.60 [2.48-12.65], *P* < .001). Overall survival did not differ between groups (hazard ratio 2.04 [0.80-5.18], *P* = .13). Cox regression demonstrated higher hazard ratios with DANE for reintervention (2.01 [1.01-3.97], *P* = .04) and reaching ≥55 mm aortic diameter (5.59 [2.33-13.42], *P* < .001).

**Conclusions and Relevance:**

DANE occurred frequently after type A aortic dissection repair and was strongly associated with accelerated downstream aortic growth and reintervention. Routine postoperative assessment and strategies aimed at preventing or treating DANE may improve long-term aortic outcomes.

## Graphical abstract

**Graphical Abstract:**
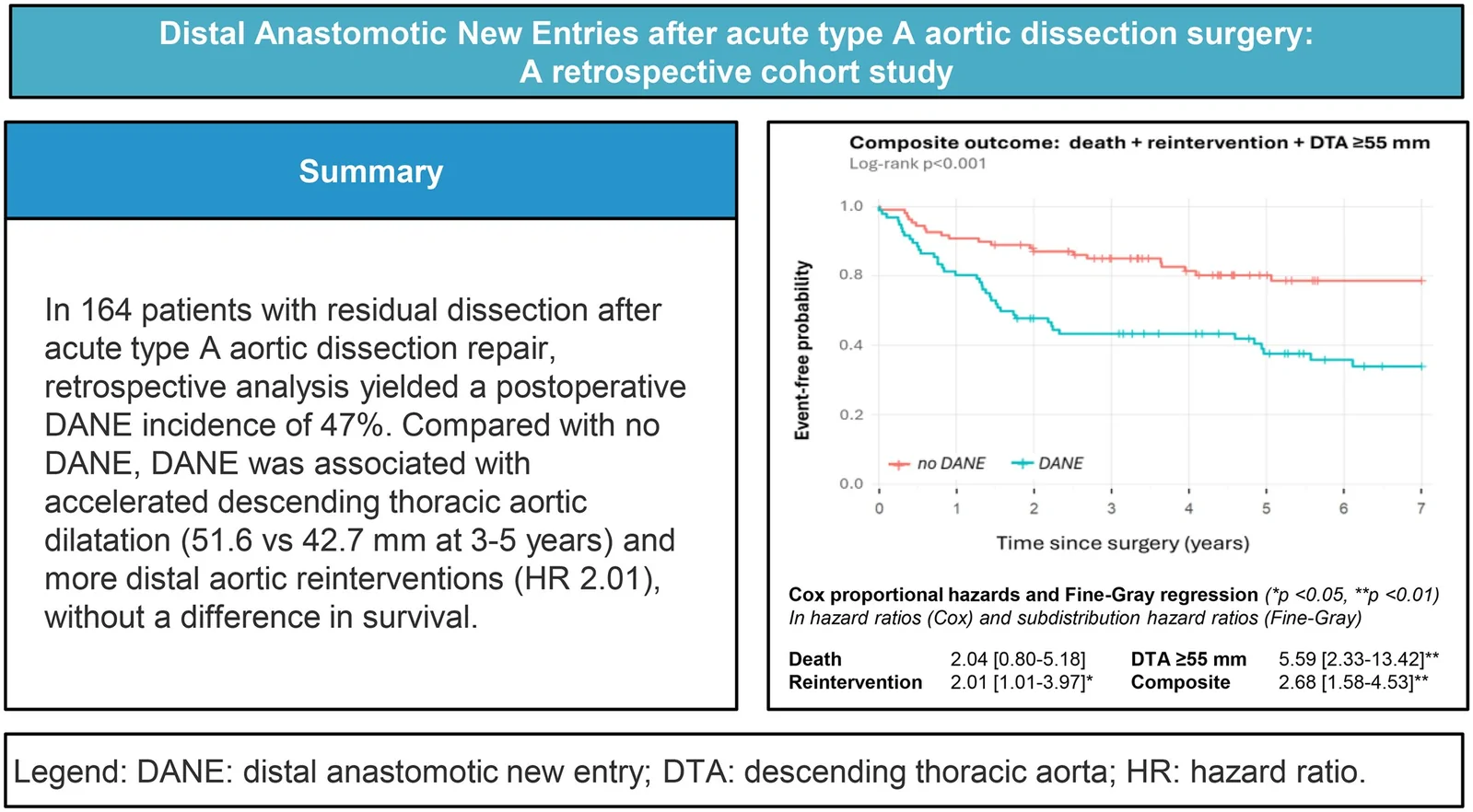

## INTRODUCTION

Acute type A aortic dissection (ATAAD) requires urgent surgical repair, yet long-term outcomes remain affected by persistent distal aortic pathology, with 10-year downstream aortic reintervention rates around 15%, and 10-year survival of 54%-65%.^1–3^ Residual false-lumen perfusion in the descending thoracic aorta (DTA) is common and is associated with progressive dilatation, increasing risk of future complications (eg, rupture) and the potential need for secondary interventions.^4^^,^^5^

Distal anastomotic new entry (DANE) is a well-recognized iatrogenic communication between the true and false lumen at the distal anastomosis site and has been reported in 20%-43% of patients after partial or complete arch repair for ATAAD.^6^^,^^7^ DANE is an important source of persistent false-lumen perfusion and has been associated with accelerated DTA growth and an increased risk of downstream aortic reintervention, with hazard ratios (HRs) exceeding 3.0 compared with patients without DANE.^8^^,^^9^ Growing awareness of the clinical consequences has increased attention to DANE prevention and detection, including the development of adjunctive devices such as the Ascyrus Medical Dissection Stent (AMDS; Artivion) and novel anastomotic devices.^6^^,^^10^ Nevertheless, the longitudinal effect of DANE on aortic diameter progression has not been adequately quantified using repeated imaging measurements over time.

This study aimed to assess the incidence of DANE and its association with longitudinal downstream aortic growth, clinically relevant dilatation, distal aortic reintervention, and survival after ATAAD repair.

## METHODS

### Study design and population

A retrospective single-centre cohort study was conducted including all consecutive patients undergoing surgical repair for ATAAD between January 2011 and December 2024. Institutional review board approval was obtained, and informed consent was waived due to the retrospective study design. Patients with residual downstream dissection (DeBakey I) and eligible CT angiography (CT-A) at least 1 year after surgery were included.

### Surgical technique

All procedures were performed using moderate to deep hypothermic circulatory arrest with selective antegrade cerebral perfusion, with the participation of at least 1 experienced aortic surgeon. The choice between hemi-arch repair and additional arch replacement was made based on the clinical situation and anatomical characteristics of the dissection. From 2022, zone 2 arch replacement was increasingly performed because of growing experience and improved options for secondary endovascular treatment.^11^ In zone 2 arch repair, the left subclavian artery generally remained in situ and was only revascularized when required by the individual anatomy and complexity of the dissection. All distal anastomoses were reinforced using external and interlaminar felt (“neo-media” technique).^12^

### Imaging and definitions

Preoperative (at the time of the dissection) and postoperative CT-A scans up to 5 years were systematically reviewed for DANE and aortic diameters. The DTA was defined as the segment extending from the left subclavian artery to the diaphragm. The extent of arch repair was defined as hemi-arch (zone 0) to zone 3 arch repair in accordance with contemporary nomenclature and definitions.^13^ Maximum aortic diameters were measured using double-oblique techniques.^14^ DANE status was determined on the first outpatient postoperative CT-A (after approximately 2-3 months), and each patient retained this classification throughout the analyses. DANE was defined as contrast opacification of the false-lumen contiguous with the prosthetic lumen at the level of the distal anastomosis (**Figure 1**). To ensure optimal visualization, the distal anastomosis was systematically evaluated using 2-plane alignment based on the external felt ring. Imaging analysis was performed by the primary investigator (S.R.v.D.) and verified by 2 board-certified interventional radiologists (J.J.J. and T.E.A.G.), both with >5 years of experience in examining aortic CT-A.

**Figure 1.**
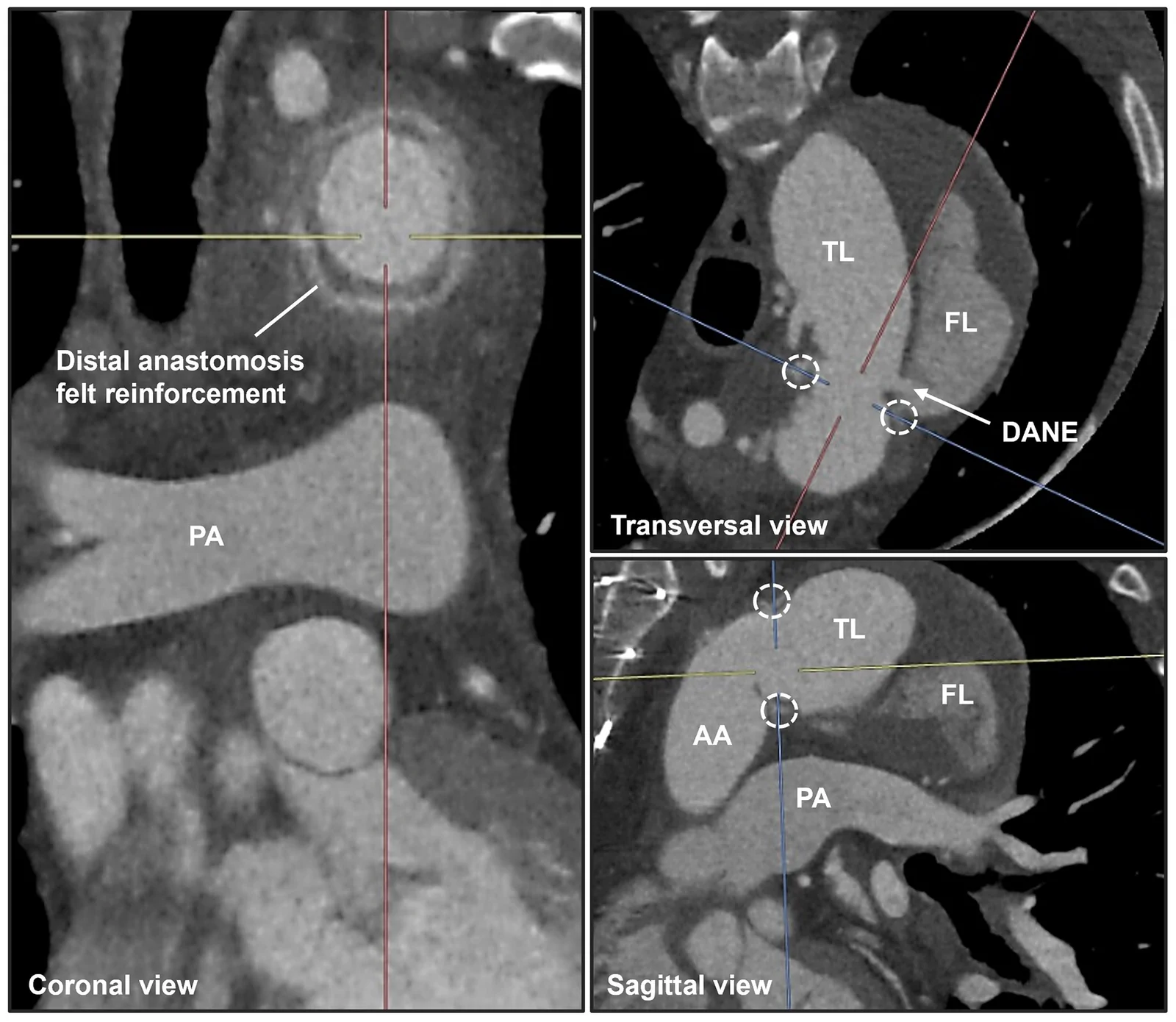
Two-Plane Alignment at the Level of the Distal Anastomosis. Multiplanar reconstruction of a CT-A scan with 2-plane alignment of the distal anastomosis, with clear DANE identification in transversal view. The dotted circles highlight the felt reinforcement used as landmarks to align the reconstruction. Abbreviations: AA, ascending aorta; FL, false lumen; PA, pulmonary artery; TL, true lumen

### Outcomes

The primary outcome was the occurrence and extent of downstream aortic growth. Downstream aortic growth was defined as the longitudinal change in maximum total DTA diameter, measured in millimetres, perpendicular to the aortic centreline (measured double oblique) on serial CT-A examinations. For the predefined interval-specific secondary outcomes, clinically relevant growth was defined as an increase of ≥5 mm at 1 year and ≥10 mm during 3- and 5-year follow-up, or attainment of a maximum DTA diameter ≥55 mm. The latter represents the institutional threshold for considering reintervention rather than an absolute treatment indication. Distal aortic reinterventions were defined as both open and endovascular procedures and were performed following multidisciplinary aortic team assessment, considering the abovementioned aortic diameter and growth rate, symptoms, anatomy, operative risk, comorbidities, and patient preference.

### Statistical analysis

Continuous variables are presented as mean ± standard deviation or median with interquartile range, as appropriate, and were compared using Student *t*-test or the Mann-Whitney *U*-test. Categorical variables are presented as counts and percentages and were compared using the chi-square or Fisher’s exact test. Univariable logistic regression analyses were performed to assess associations between DANE and the aforementioned outcome variables. To evaluate longitudinal changes in DTA diameter, linear mixed-effects models were constructed with patient-specific random intercepts, accounting for repeated measurements over time. Fixed effects included time, DANE status, and their interaction. Longitudinal analyses included all available measurements without imputation of missing imaging data. Model assumptions were assessed using residual diagnostics.

Kaplan-Meier estimates and Cox proportional hazards regression models were used to analyze overall survival and the composite end-point of death, distal aortic reintervention, or DTA diameter ≥55 mm and to estimate HRs. Proportional hazards assumptions were assessed using Schoenfeld residuals. Overall survival included all-cause mortality, irrespective of its temporal or potential causal relationship with reintervention. The cumulative incidences of distal aortic reintervention and DTA dilatation to ≥55 mm were analyzed in a competing-risk framework, with death before the respective end-point considered a competing event. Gray’s test was used for group comparisons, and Fine-Gray regression was used to estimate subdistribution HRs (sHRs). Radiological follow-up for the analysis of DTA dilatation to ≥55 mm was administratively censored at 5 years, corresponding to the predefined imaging follow-up period. The number of patients contributing data at each follow-up timepoint is reported separately.

Clinical follow-up was censored on March 1, 2026, and completeness was assessed using the Follow-Up Index. Vital status was verified for all patients at study closure or until death. Radiological follow-up was assessed separately based on eligible CT-A at 1, 3, and 5 years.

Effect estimates are reported with 95% CI and exact *P*-values where applicable, considered statistically significant when <.05. All statistical analyses were performed using R (R Foundation for Statistical Computing).

## RESULTS

Of 336 patients undergoing ATAAD repair, 164 with residual downstream dissection and eligible CT-A at 1 year were included. Clinical follow-up was complete (Follow-Up Index 1.00). Eligible imaging was available in 164 patients (100%) at 1 year, 135 (82.3%) at 3 years, and 101 (61.6%) at 5 years. Reasons for unavailable imaging are provided in **Figure S1** and **Table S1**.

Postoperative DANE was present in 77 patients (47.0%). Baseline characteristics were largely comparable (**Table 1**), with hypertension being more common in patients with DANE (55.8% vs 42.5%, *P* = .09). Patients with DANE had longer cross-clamp times (145 vs 130 min, *P* = .008) and more frequently underwent Bentall procedures (49.4% vs 32.3%, *P* = .025) and aortic valve interventions (53.2% vs 33.3%, *P* = .005). The extent of arch repair was comparable, as was DANE incidence before and from 2022 onwards (increasing number of zone 2 repair; 52/109 [47.7%] vs 25/55 [45.5%], *P* = .785). Secondary DTA re-entry tears were less frequent with DANE (18.2% vs 35.6%, *P* = .012), whereas arch vessel false-lumen involvement was comparable (39.0% vs 32.2%, *P* = .365). Distal reintervention was performed in 42 patients (25.6%), including 27 (64.3%) with DANE (*P* = .012): 26 underwent open and 16 endovascular repair, of whom 11 had previously undergone zone 2 repair.

**Table 1.** Baseline Demographic Data, Treatment Characteristics, and Follow-up Outcomes of Patients With and Without DANE After Surgical Repair of ATAAD

Variable	Total (*n* = 164)	DANE (*n* = 77)	No DANE (*n* = 87)	Unadjusted OR (95% CI)	*P*-value
**Demographics**					
Age (years)	61.0 (53.8-68.0)	61.0 (55.0-69.0)	61.0 (53.0-68.0)	1.13 (0.83-1.52)	.748
Height (cm)	178.0 (170.0-184.0)	178.5 (172.0-184.2)	176.0 (168.0-184.0)	1.02 (0.99-1.05)	.297
Weight (kg)	82.0 (70.0-94.5)	82.5 (70.0-95.2)	80.0 (69.5-93.0)	1.00 (0.98-1.02)	.653
BMI (kg/m²)	26.0 (23.9-28.9)	25.7 (23.9-29.1)	26.1 (23.9-28.7)	0.98 (0.91-1.05)	.737
GFR (mL/min/1.73 m^2^)	66.5 (55.0-81.0)	66.0 (54.0-80.0)	67.0 (55.5-83.5)	0.99 (0.98-1.01)	.515
Male sex	107 (65.6%)	53 (69.7%)	54 (62.1%)	1.41 (0.73-2.71)	.304
**Comorbidities**					
Diabetes mellitus	4 (2.4%)	3 (3.9%)	1 (1.1%)	3.49 (0.36-34.2)	.342
Atrial fibrillation	12 (7.3%)	5 (6.5%)	7 (8.0%)	0.79 (0.24-2.61)	.703
Hypertension	80 (48.8%)	43 (55.8%)	37 (42.5%)	1.71 (0.92-3.17)	.089
Hypercholesterolemia	21 (12.8%)	12 (15.6%)	9 (10.3%)	1.60 (0.63-4.03)	.316
Prior myocardial infarction	6 (3.7%)	4 (5.3%)	2 (2.3%)	2.36 (0.42-13.3)	.419
Prior TIA/CVA	15 (9.1%)	8 (10.4%)	7 (8.0%)	1.33 (0.46-3.84)	.603
Smoking history	70 (42.7%)	34 (44.2%)	36 (41.4%)	1.12 (0.60-2.08)	.720
Prior cardiac surgery	0 (0.0%)	0 (0.0%)	0 (0.0%)		
Connective tissue disorder	19 (11.6%)	9 (11.7%)	10 (11.5%)	1.02 (0.39-2.66)	.969
Bicuspid aortic valve	10 (6.3%)	7 (9.3%)	3 (3.6%)	2.78 (0.69-11.2)	.193
**Antithrombotic therapy**					
Type of therapy					.947
None	1 (0.6%)	1 (1.3%)	0 (0.0%)		
Single APT	3 (1.8%)	1 (1.3%)	2 (2.3%)		
Dual APT	53 (32.3%)	26 (33.8%)	27 (31.0%)		
OAC	28 (17.1%)	13 (16.9%)	15 (17.2%)		
OAC + SAPT	1 (0.6%)	1 (1.3%)	0 (0.0%)		
Use of any OAC	81 (49.4%)	39 (50.6%)	42 (48.3%)	1.10 (0.60-2.03)	.762
Use of any APT	108 (65.9%)	50 (64.9%)	58 (66.7%)	0.93 (0.49-1.77)	.815
**Operative characteristics**					
Extracorporeal circulation time (min)	232.0 (197.0-280.0)	237.5 (198.2-285.0)	231.0 (197.0-272.5)	1.00 (0.96-1.05)	.670
Aortic clamping time (min)	139.5 (119.0-169.5)	145.0 (131.5-184.5)	130.5 (111.0-163.5)	1.06 (1.00-1.14)	**.008**
Circulatory arrest time (min)	36.0 (29.0-46.0)	37.0 (31.0-47.2)	34.0 (29.0-41.5)	1.06 (0.89-1.25)	.126
Central cooling temp (°C)	23.0 (20.0-25.0)	23.0 (20.0-25.0)	22.0 (20.0-25.0)	1.03 (0.90-1.17)	.639
Operative technique					.184
(Frozen) elephant trunk	5 (3.0%)	0 (0.0%)	5 (5.7%)		
Hemi-arch replacement	121 (73.8%)	58 (75.3%)	63 (72.4%)		
Zone 1	4 (2.4%)	3 (3.9%)	1 (1.1%)		
Zone 2	32 (19.5%)	15 (19.5%)	17 (19.5%)		
Zone 3	2 (1.2%)	1 (1.3%)	1 (1.1%)		
Bentall procedure	66 (40.2%)	38 (49.4%)	28 (32.2%)	2.05 (1.09-3.87)	**.025**
Aortic valve intervention					**.005**
Biological	34 (20.7%)	24 (31.2%)	10 (11.5%)	Reference	
Mechanical	36 (22.0%)	17 (22.1%)	19 (21.8%)	0.37 (0.14-1.00)	
None	94 (57.3%)	36 (46.8%)	58 (66.7%)	0.26 (0.11-0.60)	
Additional procedures					.295
CABG	13 (7.9%)	5 (6.5%)	8 (9.2%)	Reference	
Left subclavian artery bypass	3 (1.8%)	0 (0.0%)	3 (3.4%)		
None	148 (90.2%)	72 (93.5%)	76 (87.4%)	1.52 (0.47-4.85)	
**Follow-up outcomes**					
Imaging characteristics					
Re-entry tear DTA	45 (27.4%)	14 (18.2%)	31 (35.6%)	0.33 (0.16-0.71)	.012
False-lumen perfusion through arch vessels	58 (35.4%)	30 (39.0%)	28 (32.2%)	1.34 (0.71-2.56)	.365
Distal aortic reintervention	42 (25.6%)	27 (35.1%)	15 (17.2%)	2.59 (1.25-5.36)	**.012**
Open surgical	26 (15.9%)	17 (22.1%)	9 (10.3%)	2.46 (1.02-5.89)	.053
Endovascular	16 (9.8%)	10 (13.0%)	6 (6.9%)	2.01 (0.70-5.83)	.292

Data are presented as median (IQR) or *n* (%). Unadjusted odds ratios describe the association with postoperative DANE and are reported per 10-year increase in age, per 10-minute increase in operative times, and per 1-unit increase for other continuous variables, unless stated otherwise. Abbreviations: APT, antiplatelet therapy; BMI, body mass index; CABG, coronary artery bypass grafting; CVA, cerebrovascular accident; DTA, descending thoracic aorta; GFR, glomerular filtration rate; OAC, oral anticoagulation; OR, odds ratio; TIA, transient ischaemic attack.

The linear mixed-effects model included 895/1148 potential imaging observations (22.0% missing), with a median follow-up of 3.63 years (IQR 2.04-4.71). DTA diameter increased over time (*P* < .001; **Figure 2A**), with significantly faster dilatation in patients with DANE (*P* < .001; **Figure 2B**). At 3-5 years, estimated mean diameters were 51.6 mm (95% CI, 49.9-53.4) with DANE and 42.7 mm (95% CI, 41.1-44.4) without DANE (*P* < .001). Residual diagnostics demonstrated mild non-normality and limited heteroscedasticity without major model-assumption violations.

**Figure 2.**
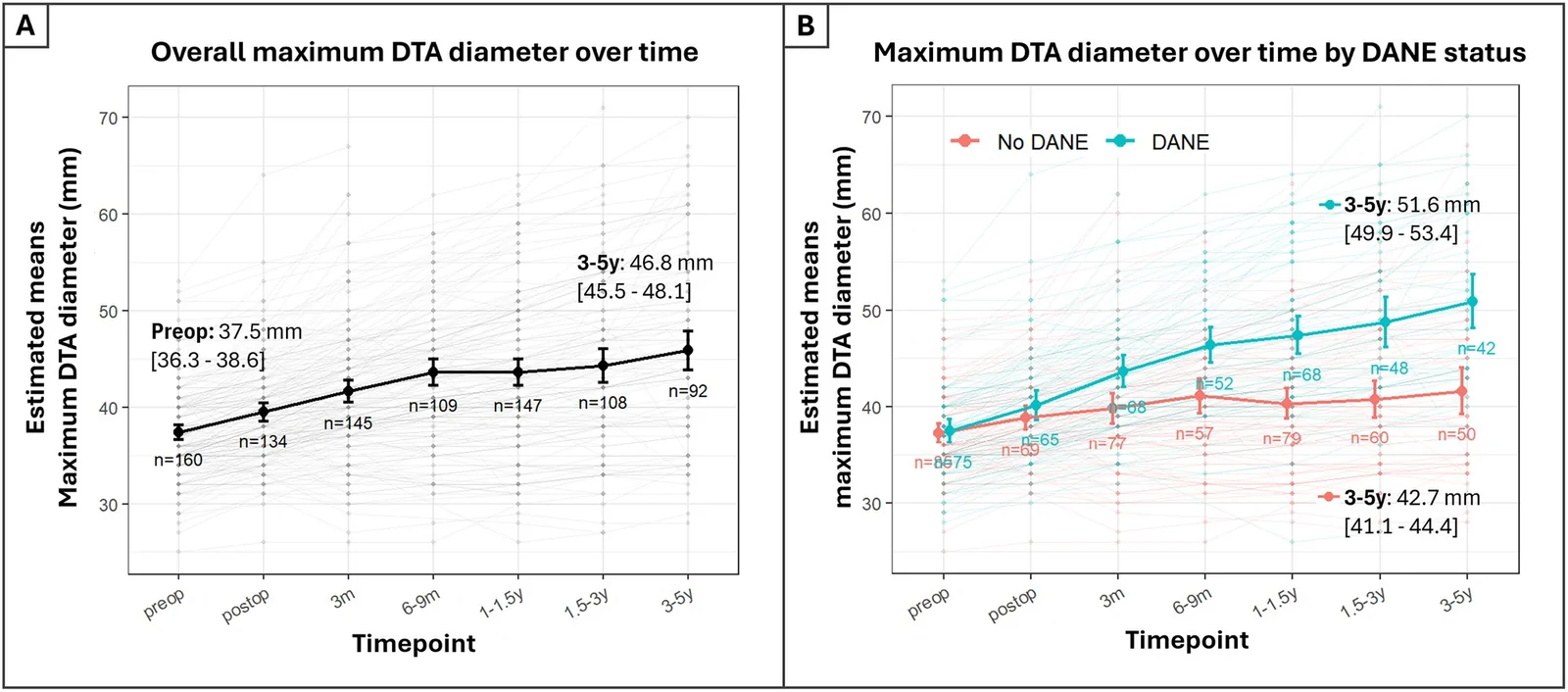
Longitudinal Changes in Maximum DTA Diameter of the Total Study Cohort and Stratified by the Presence of DANE. Linear mixed-effects model, with error bars indicating 95% CIs of all patients (**A**) and stratified per DANE status (**B**). Patients with DANE demonstrated significantly accelerated DTA growth compared with patients without DANE (time × DANE interaction *P* < .001). At 3-5 years, estimated mean DTA diameter was 51.6 mm in patients with DANE vs 42.7 mm in patients without DANE (*P* < .001)

**Figure 3** shows univariate logistic regression of the effect of DANE on adverse downstream aortic outcomes. At 1-year follow-up (*n* = 164), DANE was associated with increased odds of DTA growth ≥5 mm (OR 12.06; 95% CI, 4.67-31.14; *P* < .001), a DTA diameter ≥55 mm (OR 28.08; 95% CI, 3.63-216.99; *P* = .001) and the composite end-point of ≥5-mm growth, threshold for reintervention (≥55 mm) and reintervention (OR 11.30; 95% CI, 4.44-28.76; *P* < .001). At 3 years (*n* = 135), DANE remained significantly associated with DTA growth ≥10 mm (OR 5.98; 95% CI, 2.53-14.10; *P* < .001), DTA diameter ≥55 mm (OR 19.67; 95% CI, 2.45-157.63; *P* = .005), and the composite end-point (OR 5.72; 95% CI, 2.70-12.10; *P* < .001). No significant association between DANE and reintervention alone was observed at the 1-year and 3-year time points. At 5 years (*n* = 101), DANE remained significantly associated with DTA growth ≥10 mm (OR 6.18; 95% CI, 2.48-15.36; *P* < .001), DTA diameter ≥55 mm (OR 8.70; 95% CI, 2.31-32.81; *P* = .001), and the composite end-point (OR 5.60; 95% CI, 2.48-12.65; *P* < .001). DANE was associated with reintervention alone with borderline significance at 5-year follow-up (OR 2.18; 95% CI, 0.97-4.89; *P* = .058).

**Figure 3.**
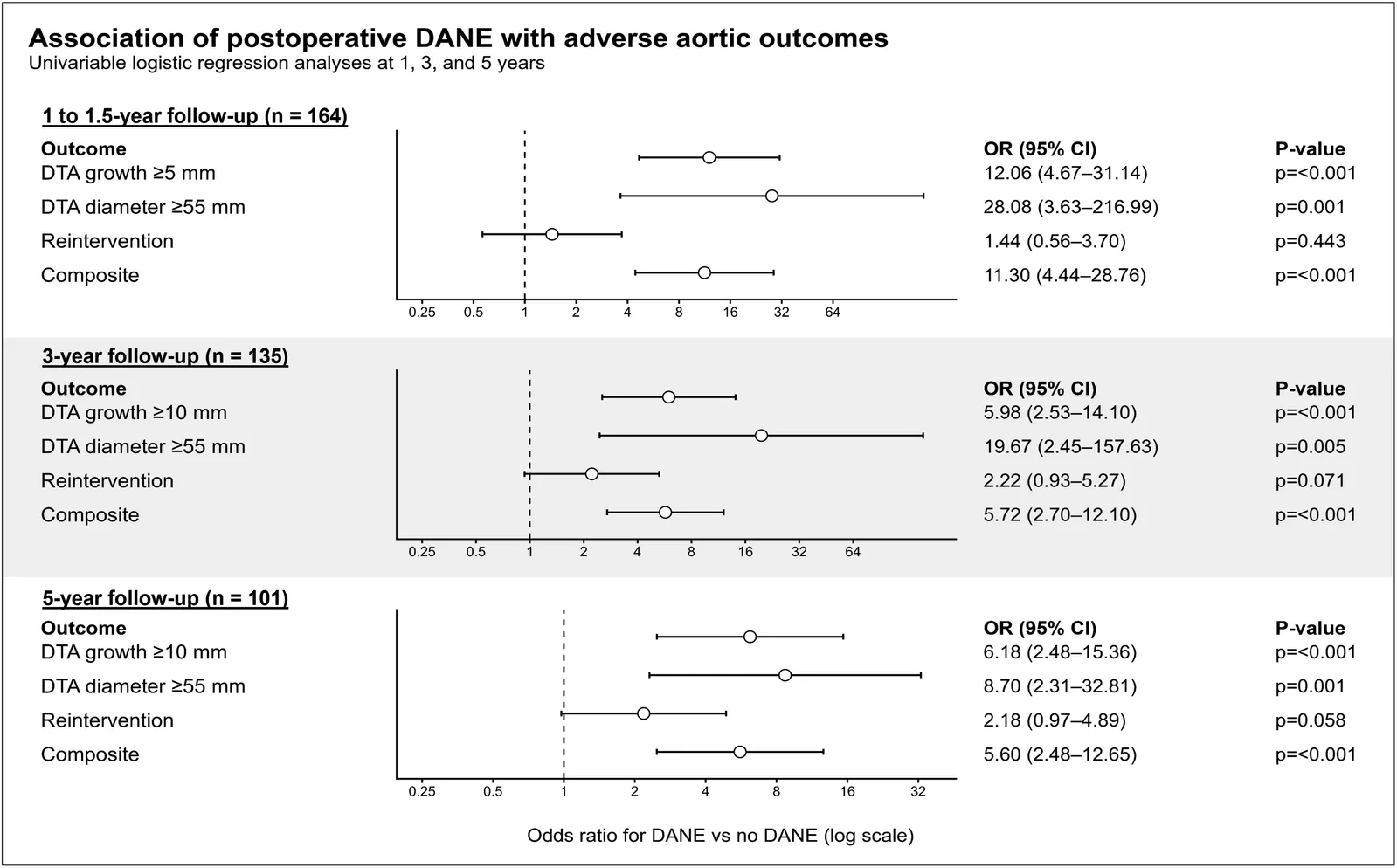
Univariable Logistic Regression Analyses of the Effect of DANE on Adverse DTA Outcomes. Forest plots demonstrate odds ratios with 95% CIs for DTA growth of ≥5 and ≥10 mm from baseline, DTA diameter ≥55 mm, distal aortic reintervention, and composite adverse aortic outcomes at 1-, 3-, and 5-year follow-up

Kaplan-Meier analysis demonstrated no difference in overall survival between patients with and without DANE (*P* = .126; **Figure 4A**), with comparable median follow-up of 4.9 and 5.2 years. DANE was associated with higher cumulative incidences of distal aortic reintervention (sHR 2.01; 95% CI, 1.01-3.97; *P* = .043) and DTA dilatation to ≥55 mm (sHR 5.59; 95% CI, 2.33-13.42; *P* < .001; **Figure 4B and C**). Freedom from the composite end-point (death, distal aortic reintervention, and DTA of ≥55 mm) was significantly lower in patients with DANE (HR 2.68; 95% CI, 1.58-4.53; *P* < .001; **Figure 4D**).

**Figure 4.**
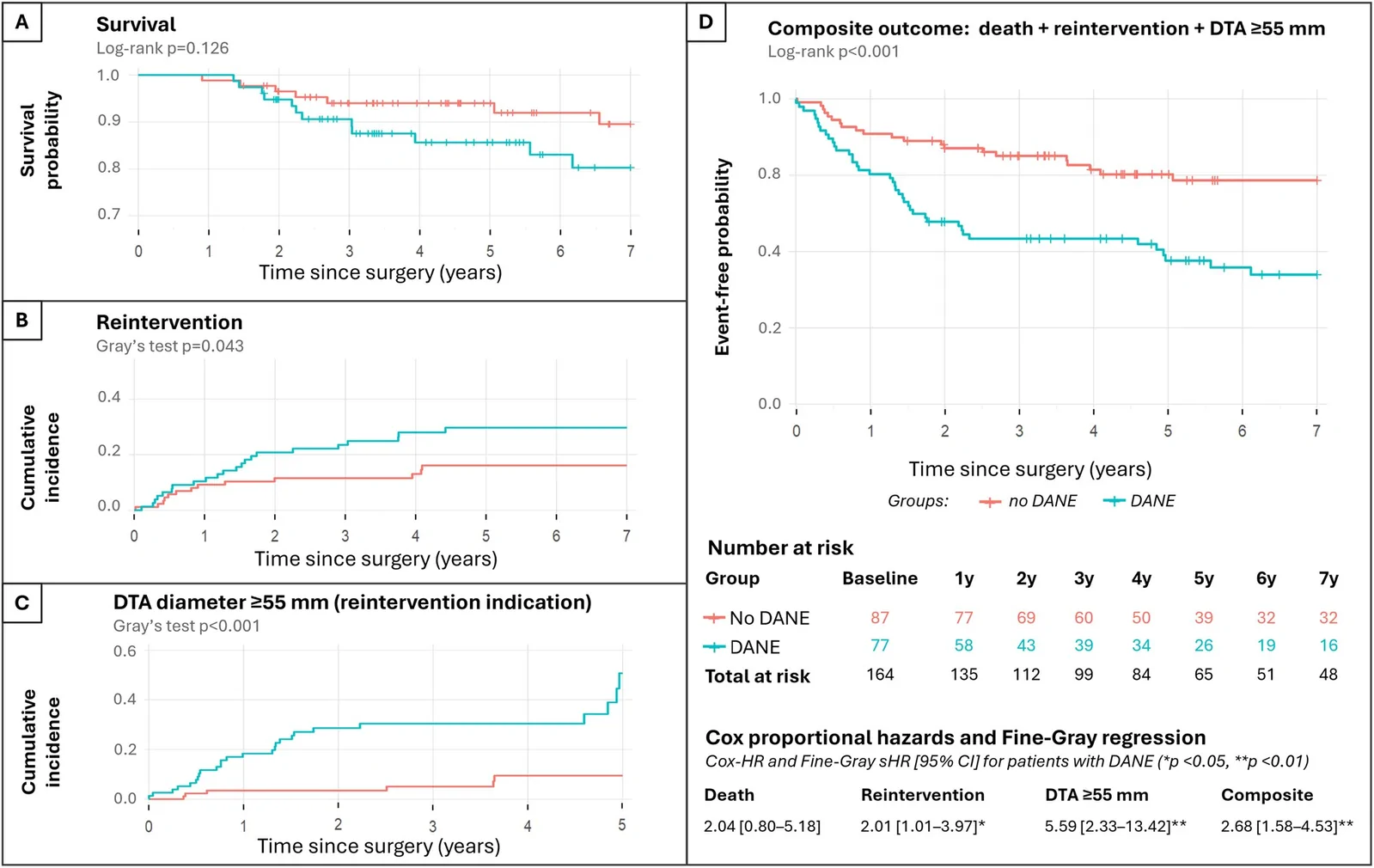
Time-to-Event Outcomes According to Postoperative DANE Status. (**A**) Kaplan-Meier estimates of overall survival. (**B**) Cumulative incidence of distal aortic reintervention and (**C**) DTA dilatation to ≥55 mm, with death considered a competing event. (**D**) Kaplan-Meier estimates of freedom from the composite end-point of death, distal aortic reintervention, or ≥55 mm DTA dilatation. HRs and sHR with 95% CIs derived from Cox proportional hazards regression and Fine-Gray’s test are shown below (**D**)

## DISCUSSION

The present study demonstrates that DANE is a frequent postoperative finding after ATAAD repair, occurring in 47% of patients, and is strongly associated with adverse distal aortic remodelling. Patients with DANE demonstrated accelerated DTA growth, higher risks of reaching the intervention threshold of ≥55 mm, and more distal aortic reinterventions. Overall survival did not differ between groups during a median follow-up of 5 years.

The association between DANE and adverse remodelling appears plausible, as persistent false-lumen inflow through the distal anastomosis may maintain false-lumen pressurization despite proximal repair. Patent false lumen has previously been associated with adverse aortic remodelling and reduced long-term survival.^5^^,^^15^ In the present longitudinal analysis, baseline diameters were comparable, but growth trajectories diverged from 3 months onwards. This supports a cumulative effect of DANE-mediated false-lumen perfusion on downstream aortic growth.

Patients with DANE more frequently had hypertension, underwent Bentall procedures, and had longer aortic cross-clamp and circulatory arrest times. Long-term hypertension may increase susceptibility to intimal disruption,^16^^,^^17^ whereas the procedural associations may reflect greater disease or operative complexity rather than directly contributing to DANE formation. Connective tissue disorders were equally distributed between groups, although their independent effect could not be assessed because of limited numbers. Furthermore, the extent of arch replacement and operative strategy were not associated with DANE, suggesting that its occurrence is not determined solely by repair complexity. Larger studies are required to distinguish patient-, anatomical-, and technique-related determinants.

The absence of a survival difference with DANE is consistent with previous reports,^7^^,^^18^ although follow-up may have been insufficient for late differences to emerge. Contemporary imaging surveillance and timely reintervention may also mitigate the effect of DANE on mortality. Nevertheless, progressive dilatation, repeated imaging, and secondary interventions impose a substantial burden on patients. Distal aortic repair may prevent aortic-related mortality but carries risks of major complications, including paraplegia and end-stage renal failure, particularly after open repair.^19^^,^^20^

Strategies to prevent DANE are therefore increasingly relevant. The AMDS combines distal intimal reinforcement with an uncovered stent in the proximal descending aorta and has been associated with false-lumen thrombosis and DANE rates of approximately 10%-17%.^6^^,^^21–23^ Conventional or frozen elephant trunk techniques may similarly support the distal anastomosis and promote false-lumen closure, although more extensive repair increases procedural complexity and may increase spinal cord ischaemia risk.^24^^,^^25^ Less extensive approaches, including optimized intimal reinforcement, may reduce DANE with lower procedural risk, but supporting evidence remains limited.

The present findings also raise the question of whether DANE should be considered a therapeutic target rather than merely an imaging finding. Early endovascular exclusion could theoretically reduce persistent false-lumen pressurization and subsequent aneurysmal degeneration. Zone 2 arch replacement may facilitate this strategy by creating a proximal landing zone for staged endovascular completion.^11^^,^^26^^,^^27^ As branched endovascular technologies evolve, such approaches may become increasingly relevant for patients at high risk of adverse remodelling, particularly those with DANE.^28^

This study has several limitations. Its retrospective, single-centre design limits causal inference and introduces potential selection bias. Surgical strategy evolved during the study period, although DANE incidence remained comparable before and after 2022. Some patients underwent reintervention before reaching 55 mm because of rapid growth; however, including these interventions reflects real-world practice. Subgroup analyses were limited by event numbers, and residual confounding by secondary re-entry tears, postoperative hypertension, and other factors cannot be excluded. Quality-of-life outcomes and detailed indications for all reinterventions were not systematically available.

Strengths include the relatively large aortic dissection cohort, systematic 2-plane assessment of the distal anastomosis, and repeated imaging analysis using linear mixed-effects modelling. These findings not only identify DANE as an important driver of adverse downstream remodelling but also stress the importance of systematic assessment of DANE during postoperative surveillance. Strategies aimed at preventing or excluding DANE may improve long-term aortic outcomes and reduce the burden of downstream dilatation and reintervention.

Collectively, these findings support DANE as an important mechanistic driver of adverse downstream aortic remodelling after ATAAD repair. Increased awareness, systematic assessment, and prevention or early exclusion of DANE may improve long-term aortic outcomes and reduce the burden of downstream aortic dilatation.

## Supplementary Material

ivag262_Supplementary_Data

## Data Availability

The data underlying this article will be shared on reasonable request to the corresponding author.
