# Supplementary material for: Distal Anastomotic New Entries After Acute Type A Aortic Dissection Surgery: A Retrospective Cohort Study
Source: Interdiscip Cardiovasc Thorac Surg. 2026 Sep 25;41(11):ivag262. doi: 10.1093/icvts/ivag262 (PMC13730567; doi:10.1093/icvts/ivag262)
Supplement: Supplementary file 1 [file ivag262_supplementary_data.docx]

# Supplementary data

## **Supplementary figure 1.** Study cohort and availability of longitudinal CT-A follow-up.

Of 336 patients undergoing acute type A aortic dissection repair, 164 (48.8%) had residual downstream dissection and were included in the study. Postoperative distal anastomotic new entry (DANE) was present in 77 patients (47.0%) and absent in 87 (53.0%). Computed tomography angiography (CT-A) was available in 164 (100%), 131 (79.9%), and 101 (61.6%) patients at 1-, 3-, and 5-year follow-up, respectively. Follow-up windows are presented independently because the absence of imaging in one window did not necessarily preclude imaging at a later timepoint.

DTA: descending thoracic aorta

Assessed for eligibility (n=337)

Excluded (n=173)

- No follow-up of ≥1 year (e.g., loss of follow-up or death <1 year; n=32)

- No DeBakey type 1 aortic dissection, n=141)

Included in analysis (n=164)

Enrolment

Excluded (n=29 )

- Death (n=11)

- DTA reintervention (n=2)
- No eligible CT-A or follow-up timepoint not reached (n=16)

1-year analysis

Included in analysis (n=135)

Excluded (n=34)

- Death (n=2)

- DTA reintervention (n=16)
- No eligible CT-A or follow-up timepoint not reached (n=16)

3-year analysis

Included in analysis (n=101)

5-year analysis

## **Supplementary table 1.** Availability of CT-A follow-up and reasons for unavailable imaging at each follow-up timepoint.

| FU timepoint | Eligible CT-A available | Deceased before FU timepoint | DTA reintervention before FU timepoint | No eligible CT-A available or FU window not yet reached |
| --- | --- | --- | --- | --- |
| 1-1.5 year | 164 (100%) | 0 | 0 | 0 |
| 3 years | 135 (82.3%) | 11 (6.7%) | 2 (1.2%) | 16 (9.8%) |
| 5 years | 101 (61.6%) | 13 (7.9%) | 18 (11.0%) | 32 (19.5%) |

Data are presented as n (%), with percentages calculated using the complete study cohort (n=164) as denominator. Categories within each follow-up (FU) timepoint are mutually exclusive. Patients undergoing downstream aortic reintervention before the respective timepoint were excluded because subsequent imaging no longer reflected the untreated course of residual aortic dissection. CT-A, computed tomography angiography; DTA, descending thoracic aorta.
